# Training Global Health Leaders: A Critical Review of Competency Gaps

**DOI:** 10.5334/aogh.3260

**Published:** 2021-07-12

**Authors:** Bhakti Hansoti, Elizabeth Hahn, Caroline Dolive, Anike Akridge, Melanie Atwell, Anant Mishra, Meike Schleiff

**Affiliations:** 1 Department of Emergency Medicine, Johns Hopkins School of Medicine, Baltimore, USA; 2 Department of International Health, Johns Hopkins Bloomberg School of Public Health, Baltimore, USA; 3 Sustaining Technical and Analytical Resources (STAR) project, Public Health Institute, USA; 4 Johns Hopkins Department of International Health, Johns Hopkins Bloomberg School of Public Health, Baltimore, USA

## Abstract

**Background:**

Global health leadership training seeks to strengthen the existing global health workforce to build leaders that have the necessary knowledge, attitudes, and skills to deliver a vision for public health and healthcare delivery. In order to develop impactful training curricula, there is a greater need to understand the areas of focus required to strengthen the global health workforce.

**Objectives:**

This paper seeks to present a critical analysis of the competency gaps among participants of a single global health training program.

**Methods:**

This is a cross-sectional observational study conducted during the implementation of the Sustaining Technical and Analytical Resources (STAR) project from May 1, 2018 to May 31, 2020. We utilized descriptive statistics to analyze the baseline competency assessment of STAR participants using a customized framework that was developed for the program.

**Findings:**

Among the 74 individuals enrolled in the study, we identified that there were significant differences in milestone achievement across participant types for all eight competencies (p < 0.001). Overall, US-based fellows reported higher perceived competency levels than low- and middle-income (LMIC)-based fellows in all categories except Capacity Strengthening (4, 23.5% leading vs. 12, 63.5% leading). LMIC fellows reported lower achieved milestones in Gender Equity (only 6, 31.5% at practicing) and Development Practice (only 6, 31.5% at practicing).

**Conclusions:**

Our study identified critical needs in the domains of public health ethics, health equity, and social justice and gender equity. Further emphasis on these domains in global health curricula and other professional development is critical to strengthen the knowledge and skills of individuals who are well-placed to advance the development of an equitable global health workforce.

## Introduction

Around the world, the shortage of human resources in the form of public health leaders and health professionals has become a prominent obstacle to sustaining health programs and improving health outcomes [1]. Effective global health leaders can play a pivotal role across a number of health initiatives from maternal and child mortality to vaccine coverage to primary care access, provided they have the appropriate technical and leadership training to make a lasting impact [1,2]. Sustained improvements in health programming require skills not only in disease-focused technical areas, but also in program management, policy making, budgeting, supply chain, financing, and many other components of public health [3,4,5,6]. The broad areas of practice of global health professionals mandate that the diversity of training they receive match the diversity of the roles they fill [3,4,5,6].

In recent decades, multiple organizations have highlighted the importance of both global health leadership programs and global health competencies to guide training activities [7]. Competency-based educational programs focus on students attaining a proficient level of practice by increasing relevant knowledge, skills, and attitudes [8]. Several organizations have used peer-review methodologies combined with consultative processes to outline a set of core competencies to support leadership development within the field of global health [7,9,10,11,12]. These have since been expanded to define scope and curricula for training programs [7,8,13,14,15,16,17].

One such global health leadership program is the Sustaining Technical and Analytic Resources (STAR) project, administered by the Public Health Institute and supported by the United States Agency for International Development (USAID). STAR is a fellowship and internship program with a focus on tailored learning opportunities for participants [18,19]. STAR participants are based in both the US (often at USAID) and in LMICs, represent a range of experience levels (1–20+ years of experience), and work across a wide range of focus areas. To inform the learning activities of STAR participants, the program built on existing competency frameworks to develop a strategy to capture the growth and training needs of participants across the program. In this paper, we seek to present the competency and milestones assessments of participants entering the STAR project to provide an insight into the training needs of global health professionals.

## Methods

This study is a cross-sectional observational study conducted during the implementation of the STAR project from May 1, 2018 to May 31, 2020. All STAR participants and interns who have completed their onboarding process (i.e., the competency assessment) during the study period were included in the sample.

### STAR competency assessment

**Figure 1:**
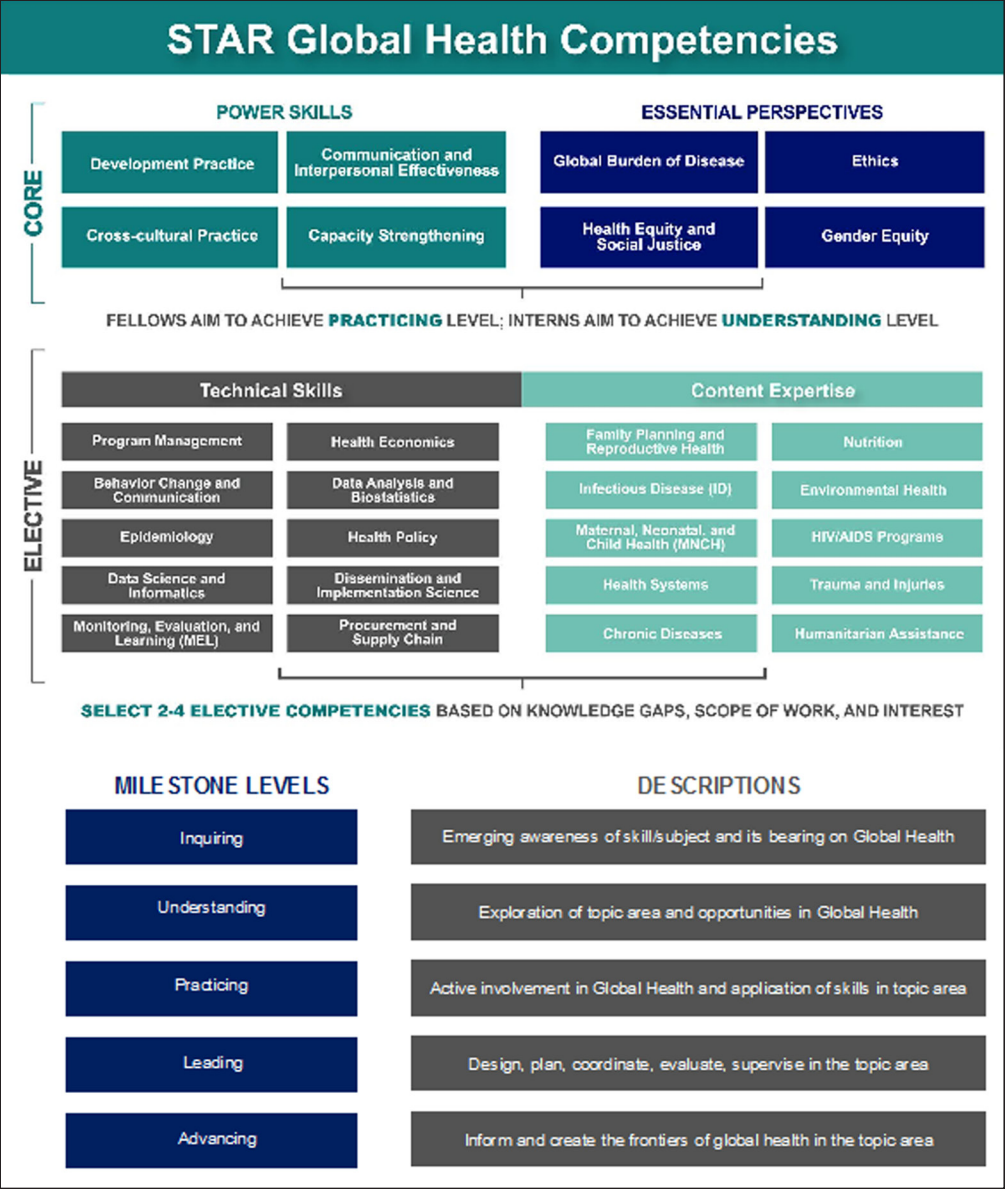
STAR Global Health Competencies and milestones.

The STAR competency framework includes eight core competency domains and 20 technical domains (both skill- and content-based) (***Figure 1***) and was informed by existing literature on global health competencies (***Figure 1***). The eight core competencies reflect four “Power Skills” (Communication and Interpersonal Effectiveness, Development Practice, Cross-Cultural Practice, and Capacity Strengthening) and four “Essential Perspectives” (Public Health Ethics, Global Burden of Disease, Gender Equity, and Health Equity and Social Justice). In addition, the STAR competency framework boosts the core competencies with elective technical competencies, which include skill domains such as Health Policy and Epidemiology and content areas like Maternal and Child Health and HIV/AIDS. For each competency domain, measurable milestones at five levels (“Inquiring,” “Understanding,” “Practicing,” “Leading,” and “Advancing”) are defined with specific roles and responsibilities for different kinds of participants. The milestones are designed to capture the full breadth of expertise and experience across participants. Under each milestone level, the framework has four knowledge- or skill-focused anchors [2]. A full description of the pedagogy of the STAR project, the learning curriculum, and the competency framework development are described in more depth elsewhere [20].

### Participants

STAR is a USAID funded fellowship program. Fellows and interns provide technical assistance and advice on USAID programs, write research papers, develop evaluation strategies and methods, and complete other tasks that enhance USAID’s operations in the sector [21]. STAR interns are required to have a master’s degree but have limited field-based experience compared to fellows who hold advanced degrees and often have more than 10 years of experience, including significant leadership roles. STAR is a unique fellowship program in that all participants received an individualized professional development plan informed by their competency assessment.

### Data Collection

Individuals onboarded to the STAR project were invited to complete a baseline competency assessment to gauge knowledge and skill levels across the STAR competency domains. The baseline competency assessment was completed using a structured interview format, which aimed to identify knowledge and skills within each participant’s prior experience that anchored individuals to a specific milestone level for each competency domain. A STAR staff person conducted each interview in order to place participants at a milestone level for each relevant competency domain. Recommended placements were checked with the participant in order to ensure that participants agreed with the staff person’s assessment and that no relevant experiences were omitted in completing the assessment. Demographic data and data regarding educational attainment and categorization, professional experience, and previous development and/or aid experience were obtained by reviewing the individual curriculum vitae for each STAR participant.

### Outcomes and data analysis

The primary outcome of this study was to determine the baseline competency for each participant across the eight core competency domains (***Figure 1***) in order to understand the baseline strengths and gaps of participants entering the STAR project. Secondary outcomes included examining the characteristics of the participants against the competency levels achieved to understand both how to better support global health professionals with a wide range of backgrounds and how to focus the development of additional training efforts. A descriptive statistical approach was taken during data analysis and was completed using Stata v.15 (StataCorp, LLC, Texas). Pearson’s chi-squared test was used to compare global health learners across various characteristics.

### Ethical Approval

Ethical approval was sought and received from the institutional review boards of the Public Health Institute (IRB #I19-022) and the Johns Hopkins Bloomberg School of Public Health (IRB00011259). Informed consent was sought in writing from all participants.

## Results

A total of 74 STAR participants completed a baseline competency assessment at the beginning of their program. Of those 74 participants, 38 (51.4%) were interns, 17 (23.0%) were US-based fellows, and 19 (25.7%) were low- and middle-income (LMIC)-based fellows (***Table 1***). The majority of interns (84.2%) and US-based fellows (58.8%) were female, while most LMIC-based fellows were male (79.0%). Fellows had higher proportions of doctoral and medical degrees, accounting for one third to one half of the degrees earned for these STAR participants, respectively. Almost 90% of STAR interns had earned at least a master’s degree prior to the start of their program. Professional experience of at least 10 years was common for fellows (76.5% US-based, 84.2% LMIC-based), but only 1 (2.6%) intern reported professional experience of this duration. Less than half (44.7%) of interns reported previous development and/or aid experience, while at least 5 years of experience was reported by 76.5% of US-based fellows and 84.2% of LMIC-based fellows.

**Table 1:** STAR participant characteristics & demographic information by participant category.


	INTERNS (n = 38)	US-BASED FELLOWS (n = 17)	LMIC-BASED FELLOWS (n = 19)	TOTAL (N = 74)

**Gender**				

Male	6 (15.8%)	7 (41.2%)	15 (79.0%)	28 (37.8%)

Female	32 (84.2%)	10 (58.8%)	4 (21.0%)	46 (62.2%)

**Degree Categories**				

Bachelor	6 (15.8%)	1 (5.9%)	2 (10.5%)	9 (12.2%)

Master of Public/Global Health	28 (73.7%)	3 (17.6%)	3 (15.8%)	34 (45.9%)

Master of Education	0 (0.0%)	1 (5.9%)	0 (0.0%)	1 (1.3%)

PhD	0 (0.0%)	4 (23.5%)	0 (0.0%)	4 (5.4%)

Medical Degree	1 (2.6%)	5 (29.4%)	7 (36.8%)	13 (17.6%)

Miscellaneous	3 (7.9%)	3 (17.6%)	6 (31.6%)	12 (16.2%)

Unknown	0 (0.0%)	0 (0.0%)	1 (5.3%)	1 (1.3%)

**Professional Experience**				

<10 years	37 (97.4%)	4 (23.5%)	3 (15.8%)	44 (59.5%)

≥10 years	1 (2.6%)	13 (76.5%)	16 (84.2%)	30 (40.5%)

**Previous Development or Aid Experience**				

None	21 (55.3%)	1 (5.9%)	2 (10.5%)	24 (32.4%)

<5 years	14 (36.8%)	3 (17.6%)	3 (15.8%)	20 (27.0%)

≥5 years	3 (7.9%)	13 (76.5%)	12 (63.2%)	28 (37.8%)

Unknown	0 (0.0%)	0 (0.0%)	2 (10.5%)	2 (2.7%)

Optional Skill Competencies*				

Project Management	3 (7.9%)	6 (35.3%)	6 (31.6%)	15 (20.3%)

Health Economics	1 (2.6%)	0 (0.0%)	2 (10.5%)	3 (4.0%)

BCC^	7 (18.4%)	2 (11.8%)	2 (10.5%)	11 (14.9%)

Data Anal. & Biostat.	10 (26.3%)	4 (23.5%)	2 (10.5%)	16 (21.6%)

Epidemiology	7 (18.4%)	0 (0.0%)	2 (10.5%)	9 (12.2%)

Health Policy	6 (15.8%)	2 (11.8%)	2 (10.5%)	10 (13.5%)

Data Sci. & Inform.	3 (7.9%)	1 (5.9%)	3 (15.8%)	7 (9.5%)

Diss. & Implement. Sci.	3 (7.9%)	4 (23.5%)	4 (21.0%)	11 (14.9%)


* STAR participants were able to select more than one competency. ^ BCC = Behavior Change & Communication. Data Anal. & Biostat. = Data Analysis & Biostatistics. Data Sci & Inform. = Data Science & Informatics. Diss. & Implement. Sci. = Dissemination & Implementation Science.

Statistically significant differences were seen in competency levels by STAR participant category across all eight core competencies (***Table 2***). A competency level of understanding was the most commonly selected level for all interns in each competency domain except Communication & Interpersonal Effectiveness, where practicing was selected by almost half of all STAR participants (36, 48.7%). There were significant differences in milestone achievement across participant types for all eight competencies (p < 0.001). Overall US-based fellows reported higher perceived competency levels than LMIC-based fellows in all categories except Capacity Strengthening (4, 23.5% leading vs. 12, 63.5% leading). LMIC fellows as a cohort overall reported lower achieved milestones in Gender Equity (6, 31.5% at practicing) and Development Practice (6, 31.5% at practicing). Additionally, no US- or LMIC-based fellows reported a competency level of advancing for the Public Health Ethics and Health Equity & Social Justice core competencies.

**Table 2:** STAR participant milestones level, across the eight core competency levels by participant category.


LEARNER CLASSIFICATION	INQUIRING	UNDERSTANDING	PRACTICING	LEADING	ADVANCING	CHI SQUARED (p-VALUE)

DEVELOPMENT PRACTICE						

Intern	3 (7.9%)	**28 (73.7%)**	7 (18.4%)	*0 (0.0%)*	*0 (0.0%)*	<0.001*

US-F	*0 (0.0%)*	4 (23.5%)	**6 (35.3%)**	**6 (35.3%)**	1 (5.9%)	

LMIC-F	2 (10.5%)	**10 (52.6%)**	6 (31.6%)	*0 (0.0%)*	1 (5.3%)	

Total	5 (6.8%)	**42 (56.8%)**	19 (25.7%)	6 (8.1%)	*2 (2.7%)*	

**COMMUNICATION & INTERPERSONAL EFFECTIVENESS**						

Intern	2 (5.3%)	**22 (57.9%)**	14 (36.8%)	*0 (0.0%)*	*0 (0.0%)*	<0.001*

US-F	*0 (0.0%)*	*0 (0.0%)*	6 (35.3%)	**10 (58.8%)**	1 (5.9%)	

LMIC-F	*0 (0.0%)*	*0 (0.0%)*	**16 (84.2%)**	2 (10.5%)	1 (5.3%)	

Total	*2 (2.7%)*	22 (29.7%)	**36 (48.7%)**	12 (16.2%)	*2 (2.7%)*	

**CROSS-CULTURAL PRACTICE**						

Intern	6 (15.8%)	**24 (63.2%)**	8 (21.0%)	*0 (0.0%)*	*0 (0.0%)*	<0.001*

US-F	*0 (0.0%)*	1 (5.9%)	4 (23.5%)	**10 (58.8%)**	2 (11.8%)	

LMIC-F	*0 (0.0%)*	4 (21.0%)	**12 (63.2%)**	3 (15.8%)	*0 (0.0%)*	

Total	6 (8.1%)	**29 (39.2%)**	24 (32.4%)	13 (17.6%)	*2 (2.7%)*	

**CAPACITY STRENGTHENING**						

Intern	9 (23.7%)	**23 (60.5%)**	6 (15.8%)	*0 (0.0%)*	*0 (0.0%)*	<0.001*

US-F	*0 (0.0%)*	1 (5.9%)	**11 (64.7%)**	4 (23.5%)	1 (5.9%)	

LMIC-F	*0 (0.0%)*	*0 (0.0%)*	7 (36.8%)	**12 (63.2%)**	*0 (0.0%)*	

Total	9 (12.2%)	**24 (32.4%)**	**24 (32.4%)**	16 (21.6%)	*1 (1.4%)*	

**GLOBAL BURDEN OF DISEASE**						

Intern	5 (13.2%)	**27 (71.0%)**	6 (15.8%)	*0 (0.0%)*	*0 (0.0%)*	<0.001*

US-F	*0 (0.0%)*	1 (5.9%)	6 (35.3%)	**8 (47.1%)**	2 (11.8%)	

LMIC-F	*0 (0.0%)*	2 (10.5%)	**11 (57.9%)**	5 (26.3%)	1 (5.3%)	

Total	5 (6.8%)	**30 (40.5%)**	23 (31.1%)	13 (17.6%)	*3 (4.0%)*	

**ETHICS**						

Intern	8 (21.1%)	**29 (76.3%)**	1 (2.6%)	*0 (0.0%)*	*0 (0.0%)*	<0.001*

US-F	*0 (0.0%)*	6 (35.3%)	**7 (41.2%)**	4 (23.5%)	*0 (0.0%)*	

LMIC-F	3 (15.8%)	**8 (42.1%)**	**8 (42.1%)**	*0 (0.0%)*	*0 (0.0%)*	

Total	11 (14.9%)	**43 (58.1%)**	16 (21.6%)	4 (5.4%)	*0 (0.0%)*	

**HEALTH EQUALITY & SOCIAL JUSTICE**						

Intern	7 (18.4%)	**23 (60.5%)**	8 (21.1%)	*0 (0.0%)*	*0 (0.0%)*	0.001**

US-F	1 (5.9%)	3 (17.7%)	**9 (52.9%)**	4 (23.5%)	*0 (0.0%)*	

LMIC-F	1 (5.3%)	6 (31.6%)	**11 (57.9%)**	1 (5.3%)	*0 (0.0%)*	

Total	9 (12.2%)	**32 (43.2%)**	28 (37.8%)	5 (6.8%)	*0 (0.0%)*	

**GENDER EQUITY**						

Intern	3 (7.9%)	**28 (73.7%)**	7 (18.4%)	*0 (0.0%)*	*0 (0.0%)*	<0.001*

US-F	*0 (0.0%)*	4 (23.5%)	**6 (35.3%)**	**6 (35.3%)**	1 (5.9%)	

LMIC-F	2 (10.5%)	**10 (52.6%)**	6 (31.6%)	*0 (0.0%)*	1 (5.3%)	

Total	5 (6.8%)	**42 (56.8%)**	19 (25.7%)	6 (8.1%)	*2 (2.7%)*	


Cells in italic signify the highest value. Cells in bold signify the lowest value. * Designates significance at the p < 0.001 level. ** Designates significance at the p < 0.05 level.

**Figure 2:**
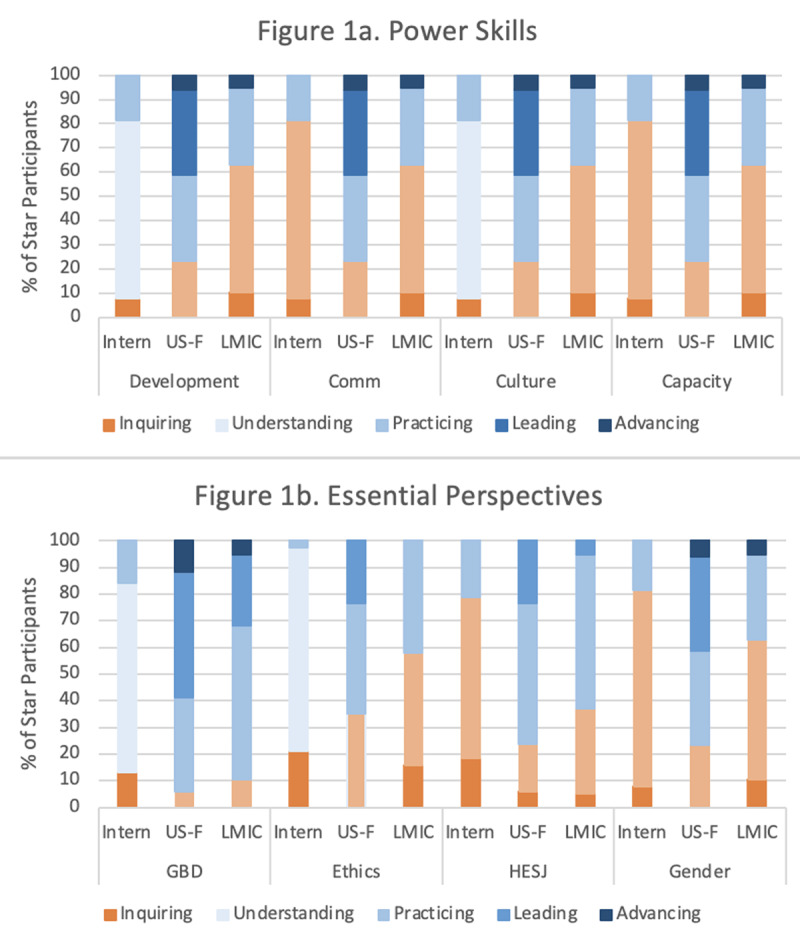
STAR participant milestones level, across the eight core competency levels by participant category*. *Shades of orange indicate proportions of STAR participants that had deficits (as defined by the program—minimum of “understanding” level for interns and minimum of “practicing” for fellow) at baseline. Shades of blue indicate adequate skill levels as defined for each type of participant; darker blue indicates higher skill levels, with the darkest blue indicating “advancing” level.

***Figure 2*** provides a visual representation of the data presented in ***Table 2*** as well. The shades of orange indicate areas of competency gaps, which can be seen most consistently among LMIC fellows for the Power Skills competencies and among interns and LMIC fellows for the Essential Perspectives competencies.

As shown in Table 3 and Figure 3 (Appendix A), all skill competencies were selected by at least one STAR participant. The top optional skill competencies selected by STAR participants differed for each participant group. Data Analysis & Biostatistics was the most common selection for interns (10, 26.3%). More than 30 percent of both US- (6, 35.5%) and LMIC-based (6, 31.6%) fellows selected Project Management in addition to their core competency selections. The skill competencies of Data Analysis & Biostatistics (p = 0.029) and Dissemination & Implementation Sciences (p = 0.012) were the only skill competencies to show significant differences in competency level by STAR participant category. In both of these skill competencies, the majority of interns reported a competency level of understanding, while US-based fellows reported either practicing or leading and LMIC-based fellows all reported a competency level of practicing.

As shown in Table 4 and Figure 4 (Appendix B), of the 10 content expertise competencies, only Environmental Health and Trauma & Injuries were never selected by STAR participants. Infectious Disease (19, 25.7%) and HIV/AIDS (17, 23.0%) were the most frequently selected competencies, while Chronic Diseases and Humanitarian Assistance were both only selected once (both times by a STAR intern; 1.4%). There were no statistically significant differences in content expertise competency levels by participant group. A competency level of advancing was not reported for any content expertise competency, and a competency level of inquiring was reported almost exclusively by interns (a single US-based fellow reported an inquiring competency level for Infectious Diseases). Interns appear to have interests differing from fellows, considering the competencies with highest frequency of intern interest being HIV/AIDS (11, 28.9%), Family Planning & Reproductive Health (6, 15.8%), and Maternal, Neonatal, & Child Health (4, 10.5%). In contrast, of the 36 total fellows, only 6 selected HIV/AIDS (16.7%), 2 selected Family Planning & Reproductive Health (5.5%), and 1 selected Maternal, Neonatal, & Child Health (2.8%). Additionally, a total of 18 fellows (6 US-based and 12 LMIC-based; 50.0%) selected the Infectious Disease competency, while only 1 intern (2.6%) selected the same competency.

## Discussion

Overall, we were excited to see that a majority of our fellows did score well on the “Power Skills” competencies essential for global health leadership, but we were surprised that many of our fellows lacked the “Essential Perspectives” that are required to inform effective leadership. Over the past 10 years, USAID has increasingly placed emphasis on its Journey to Self-Reliance initiative, which aims to strengthen a country’s own health governing capacity [21]. Accelerating a country’s Journey to Self-Reliance requires country leadership to be able to effectively marshal and manage its own development resources by building commitment and capacity. This involves building capacity not only by strengthening in-country partnerships in both the public and private sector, but also via training initiatives for program staff both in the US and abroad [22]. Given that the majority of our participants have previously worked at USAID, it is unsurprising that they were able to clearly articulate the knowledge and skills required to support capacity strengthening and impact within health programs.

Conversely, despite—or possibly partly due to—the significant in-field experience of our participants, we noted critical competencies deficits under the Essential Perspectives domains of ethics, health equity, and social justice and gender equity. It was surprising that there were deficits in the area of public health ethics, especially given that the majority of post-graduate training programs and global health leadership programs often include a focus on the ethical implications of global health practices and public health ethics. This deficit may be partly due to the fact that an explicit focus on public health ethics is a more recent development, and some of the most experienced fellows may have completed their training prior to global health curricula including these topics consistently. However, an understanding of public health ethics is vital to identify programs and interventions that are both sustainable and impactful [23]. It is critical that global health leaders are able to both identify biases when developing priorities and are able to understand the impact of an initiative, not only on the immediate beneficiary, but also on communities and health ecosystems [24,25].

In addition to the deficit in ethics, STAR participants also seemingly lacked background on equity, both gender equity and health disparities. Applications of gender equity in global health programming fall on a spectrum from being able to apply a gender lens to data and monitoring and evaluation to leading change within institutions to develop integrated gender transformative programs. Issues of equity and health disparities also inform the concept of intersectionality, the idea that relationships and interactions between age, gender, and race across multiple levels of society determine how health is distributed across demographic and geographical contexts [26]. Recognizing and acknowledging biases such as gender and race is crucial; however, to transform the global health landscape and build an equitable workforce, there needs to be a renewed focus on cultivating these essential perspectives required to develop transformative programs championed by global health leaders.

### Limitations

A factor that likely influenced the results of the skill and content expertise-based competencies of the STAR program is the potential of selection bias in the STAR participant selection process. The process is like many other staffing mechanisms where participant placements were based on host organization needs [23,27]. Hiring managers from field offices at the host organization were required to consult with STAR staff on the specific personnel needed in their respective offices. During this initial consultation, specific job duties and required skillsets were determined for each STAR placement and a scope of work was developed. Furthermore, a majority of STAR participants were placed in positions under the Global Health Bureau (GH) at USAID. Currently, the Global Health Bureau prioritizes programs focused on combating infectious diseases, preventing child and maternal deaths, and controlling the HIV/AIDS epidemic. These areas of program focus can explain the high proportion of STAR fellows placed in LMICs with a competency level of practicing or above in Infectious Disease, while no STAR participants, including LMIC fellows, exhibited Environmental Health skills at any competency-level. Thus, due to the selection of STAR participants based on program need, and the fact that most STAR participants’ positions supported the aforementioned USAID-GH programs, our study population may not be representative of global health professionals with other areas of expertise. Lastly, the competency model that was used for the evaluation was built from a broad range of exciting competency frameworks both in the peer review and grey-literature; the authors do note however that none of these frameworks were developed with LMIC-fellows in mind and thus may not reflect the experience and education of the LMIC participants [2].

## Conclusion

Despite STAR participants boasting significant technical competencies and in-field experience, many lacked the “essential perspectives”—public health ethics and equity in terms of health disparities and gender—and associated skills to champion transformative change within their organizations and the health programs they advise and lead. Further emphasis on these domains in global health curricula and other professional development is critical to strengthen the capacity of individuals who are well-placed to advance the development of an equitable global health workforce.

## Additional Files

The additional files for this article can be found as follows:

10.5334/aogh.3260.s1 Appendix A. STAR participant skill competency levels by participant category (table and graph).

10.5334/aogh.3260.s2 Appendix B. STAR participant content competency levels by participant category (table and graph).

## Author Note

The Sustaining Technical and Analytic Resources (STAR) project is a five-year cooperative agreement funded by the United States Agency for International Development Global Health Bureau (USAID/GHB), beginning May 1, 2018. It is implemented by the Public Health Institute, in collaboration with Johns Hopkins University, University of California, San Francisco, and the Consortium of Universities for Global Health. Contents are the responsibility of the STAR project, Johns Hopkins University, and the Public Health Institute and do not necessarily reflect the views of USAID or the U.S. Government.
