# Supplementary material for: Training Global Health Leaders: A Critical Review of Competency Gaps
Source: Ann Glob Health. 2021 Jul 12;87(1):65. doi: 10.5334/aogh.3260 (PMC8284503; doi:10.5334/aogh.3260)
Supplement: Appendix A. — STAR participant skill competency levels by participant category (table and graph). [file agh-87-1-3260-s1.pdf]

Appendices

Appendix A

Table 3. STAR participant skill competency levels by participant category

	Inquiring (n = 0)	*Understanding (n = 0)*	*Practicing (n = 6)*	*Leading (n = 7)*	*Advancing (n = 2)*	*Total (N = 15)*	*Chi squared p-value*
**Project Management**							
Intern	0 (0.0%)	0 (0.0%)	2 (66.7%)	1 (33.3%)	0 (0.0%)	3	
US-F	0 (0.0%)	0 (0.0%)	1 (16.7%)	3 (50.0%)	2 (33.3%)	6	0.326
LMIC-F	0 (0.0%)	0 (0.0%)	3 (50.0%)	3 (50.0%)	0 (0.0%)	6	
**Health Economics**							
	Inquiring (n = 0)	*Understanding (n = 1)*	*Practicing (n = 2)*	*Leading (n = 0)*	*Advancing (n = 0)*	*Total (N = 3)*	*Chi squared p-value*
Intern	0 (0.0%)	0 (0.0%)	1 (100.0%)	0 (0.0%)	0 (0.0%)	1	
US-F	0 (0.0%)	0 (0.0%)	0 (0.0%)	0 (0.0%)	0 (0.0%)	0	0.386
LMIC-F	0 (0.0%)	1 (50.0%)	1 (50.0%)	0 (0.0%)	0 (0.0%)	2	
**Behavioral Change & Communication**							
	Inquiring (n = 0)	*Understanding (n = 4)*	*Practicing (n = 6)*	*Leading (n = 1)*	*Advancing (n = 0)*	*Total (N = 11)*	*Chi squared p-value*
Intern	0 (0.0%)	4 (57.1%)	3 (42.9%)	0 (0.0%)	0 (0.0%)	7	
US-F	0 (0.0%)	0 (0.0%)	1 (50.0%)	1 (50.0%)	0 (0.0%)	2	0.102
LMIC-F	0 (0.0%)	0 (0.0%)	2 (100.0%)	0 (0.0%)	0 (0.0%)	2	
**Data Analysis & Biostatistics**							
	Inquiring (n = 0)	*Understanding (n = 6)*	*Practicing (n = 8)*	*Leading (n = 2)*	*Advancing (n = 0)*	*Total (N = 16)*	*Chi squared p-value*
Intern	0 (0.0%)	6 (60.0%)	4 (40.0%)	0 (0.0%)	0 (0.0%)	10	
US-F	0 (0.0%)	0 (0.0%)	2 (50.0%)	2 (50.0%)	0 (0.0%)	4	0.029*
LMIC-F	0 (0.0%)	0 (0.0%)	2 (100.0%)	0 (0.0%)	0 (0.0%)	2	
**Epidemiology**							
	Inquiring (n = 1)	*Understanding (n = 5)*	*Practicing (n = 3)*	*Leading (n = 0)*	*Advancing (n = 0)*	*Total (N = 9)*	*Chi squared p-value*
Intern	1 (14.3%)	5 (71.4%)	1 (14.3%)	0 (0.0%)	0 (0.0%)	7	
US-F	0 (0.0%)	0 (0.0%)	0 (0.0%)	0 (0.0%)	0 (0.0%)	0	0.076
LMIC-F	0 (0.0%)	0 (0.0%)	2 (100.0%)	0 (0.0%)	0 (0.0%)	2	
**Health Policy**							
	Inquiring (n = 1)	*Understanding (n = 5)*	*Practicing (n = 2)*	*Leading (n = 2)*	*Advancing (n = 0)*	*Total (N = 10)*	*Chi squared p-value*
Intern	1 (16.7%)	5 (83.3%)	0 (0.0%)	0 (0.0%)	0 (0.0%)	6	
US-F	0 (0.0%)	0 (0.0%)	1 (50.0%)	1 (50.0%)	0 (0.0%)	2	0.125
LMIC-F	0 (0.0%)	0 (0.0%)	1 (50.0%)	1 (50.0%)	0 (0.0%)	2	
**Data Science & Informatics**							
	Inquiring (n = 1)	*Understanding (n = 1)*	*Practicing (n = 2)*	*Leading (n = 3)*	*Advancing (n = 0)*	*Total (N = 7)*	*Chi squared*

							p-value
Intern	1 (33.3%)	0 (0.0%)	2 (66.7%)	0 (0.0%)	0 (0.0%)	3	0.255
US-F	0 (0.0%)	0 (0.0%)	0 (0.0%)	1 (100.0%)	0 (0.0%)	1	
LMIC-F	0 (0.0%)	1 (33.3%)	0 (0.0%)	2 (66.7%)	0 (0.0%)	3	
**Dissemination & Implementation Science**							
	Inquiring (n = 0)	*Understanding (n = 4)*	*Practicing (n = 5)*	*Leading (n = 0)*	*Advancing (n = 0)*	*Total (N = 11)*	*Chi squared p-value*
Intern	0 (0.0%)	3 (100.0%)	0 (0.0%)	0 (0.0%)	0 (0.0%)	3	0.012*
US-F	0 (0.0%)	1 (25.0%)	1 (25.0%)	2 (50.0%)	0 (0.0%)	4	
LMIC-F	0 (0.0%)	0 (0.0%)	4 (100.0%)	0 (0.0%)	0 (0.0%)	4	

Cells highlighted in green signify the highest value
*Designates significance at the $p < 0.05$ level

Figure 3: STAR participant milestones level, across the skill competency levels by participant category*

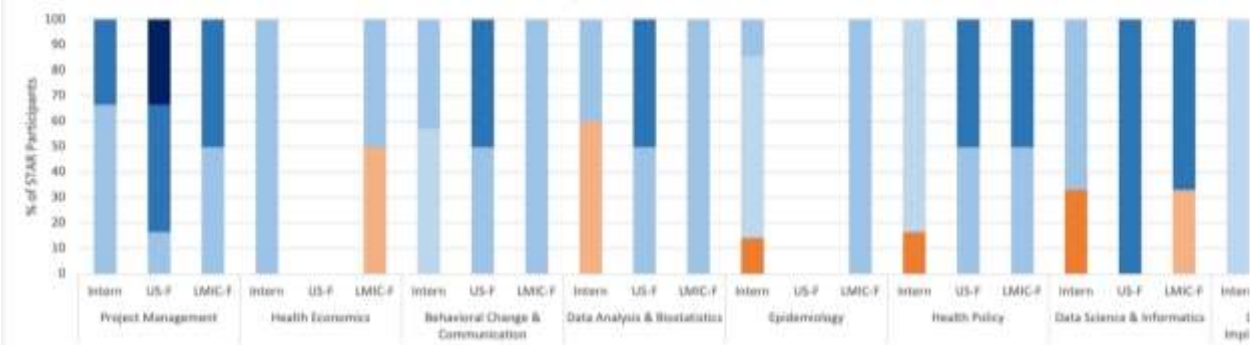

*Shades of orange indicate proportions of STAR participants that had deficits (as defined by the program—minimum of "understanding" level for interns and minimum of "practicing" for fellow) at baseline. Shades of blue indicate adequate skill levels as defined for each type of participant; darker blue indicates higher skill levels, with the darkest blue indicating "advancing" level.
