# Supplementary material for: Training Global Health Leaders: A Critical Review of Competency Gaps
Source: Ann Glob Health. 2021 Jul 12;87(1):65. doi: 10.5334/aogh.3260 (PMC8284503; doi:10.5334/aogh.3260)
Supplement: Appendix B. — STAR participant content competency levels by participant category (table and graph). [file agh-87-1-3260-s2.pdf]

Appendix B

Table 4. STAR participant content expertise competency levels by participant category

**Family Planning & Reproductive Health**							
	Inquiring (n = 1)	*Understanding (n = 2)*	*Practicing (n = 4)*	*Leading (n = 1)*	*Advancing (n = 0)*	*Total (N = 8)*	*Chi squared p-value*
Intern	1 (16.7%)	2 (33.3%)	3 (50.0%)	0 (0.0%)	0 (0.0%)	6	0.174
US-F	0 (0.0%)	0 (0.0%)	0 (0.0%)	1 (100.0%)	0 (0.0%)	1	
LMIC-F	0 (0.0%)	0 (0.0%)	1 (100.0%)	0 (0.0%)	0 (0.0%)	1	
**Nutrition**							
	Inquiring (n = 1)	*Understanding (n = 2)*	*Practicing (n = 0)*	*Leading (n = 2)*	*Advancing (n = 0)*	*Total (N = 5)*	*Chi squared p-value*
Intern	1 (33.3%)	2 (66.7%)	0 (0.0%)	0 (0.0%)	0 (0.0%)	3	0.082
US-F	0 (0.0%)	0 (0.0%)	0 (0.0%)	2 (100.0%)	0 (0.0%)	2	
LMIC-F	0 (0.0%)	0 (0.0%)	0 (0.0%)	0 (0.0%)	0 (0.0%)	0	
**Infectious Disease**							
	Inquiring (n = 1)	*Understanding (n = 1)*	*Practicing (n = 12)*	*Leading (n = 5)*	*Advancing (n = 0)*	*Total (N = 19)*	*Chi squared p-value*
Intern	0 (0.0%)	0 (0.0%)	1 (100.0%)	0 (0.0%)	0 (0.0%)	1	0.418
US-F	1 (16.7%)	0 (0.0%)	2 (33.3%)	3 (50.0%)	0 (0.0%)	6	
LMIC-F	0 (0.0%)	1 (8.3%)	9 (75.0%)	2 (16.7%)	0 (0.0%)	12	
**Maternal, Neonatal, & Child Health**							
	Inquiring (n = 2)	*Understanding (n = 2)*	*Practicing (n = 0)*	*Leading (n = 1)*	*Advancing (n = 0)*	*Total (N = 5)*	*Chi squared p-value*
Intern	2 (50.0%)	2 (50.0%)	0 (0.0%)	0 (0.0%)	0 (0.0%)	4	0.082
US-F	0 (0.0%)	0 (0.0%)	0 (0.0%)	1 (100.0%)	0 (0.0%)	1	
LMIC-F	0 (0.0%)	0 (0.0%)	0 (0.0%)	0 (0.0%)	0 (0.0%)	0	
**HIV/AIDS**							
	Inquiring (n = 0)	*Understanding (n = 8)*	*Practicing (n = 6)*	*Leading (n = 3)*	*Advancing (n = 0)*	*Total (N = 17)*	*Chi squared p-value*
Intern	0 (0.0%)	7 (63.6%)	4 (36.4%)	0 (0.0%)	0 (0.0%)	11	0.050
US-F	0 (0.0%)	1 (20.0%)	2 (40.0%)	2 (40.0%)	0 (0.0%)	5	
LMIC-F	0 (0.0%)	0 (0.0%)	0 (0.0%)	1 (100.0%)	0 (0.0%)	1	
**Health Systems**							
	Inquiring (n = 0)	*Understanding (n = 2)*	*Practicing (n = 0)*	*Leading (n = 1)*	*Advancing (n = 0)*	*Total (N = 3)*	*Chi squared p-value*
Intern	0 (0.0%)	2 (100.0%)	0 (0.0%)	0 (0.0%)	0 (0.0%)	2	0.083
US-F	0 (0.0%)	0 (0.0%)	0 (0.0%)	0 (0.0%)	0 (0.0%)	0	
LMIC-F	0 (0.0%)	0 (0.0%)	0 (0.0%)	1 (100.0%)	0 (0.0%)	1	
**Chronic Diseases**							
	Inquiring (n = 1)	*Understanding (n = 0)*	*Practicing (n = 0)*	*Leading (n = 0)*	*Advancing (n = 0)*	*Total (N =1)*	*Chi squared p-value*

Intern	1 (100.0%)	0 (0.0%)	0 (0.0%)	0 (0.0%)	0 (0.0%)	1	
US-F	0 (0.0%)	0 (0.0%)	0 (0.0%)	0 (0.0%)	0 (0.0%)	0	–
LMIC-F	0 (0.0%)	0 (0.0%)	0 (0.0%)	0 (0.0%)	0 (0.0%)	0	
**Humanitarian Assistance**							
	Inquiring (n = 1)	*Understanding (n = 0)*	*Practicing (n = 0)*	*Leading (n = 0)*	*Advancing (n = 0)*	*Total (N =1)*	*Chi squared p-value*
Intern	1 (100.0%)	0 (0.0%)	0 (0.0%)	0 (0.0%)	0 (0.0%)	1	
US-F	0 (0.0%)	0 (0.0%)	0 (0.0%)	0 (0.0%)	0 (0.0%)	0	–
LMIC-F	0 (0.0%)	0 (0.0%)	0 (0.0%)	0 (0.0%)	0 (0.0%)	0	

Cells highlighted in green signify the highest value

Figure 4: STAR participant milestones level, across the content competency levels by participant category*

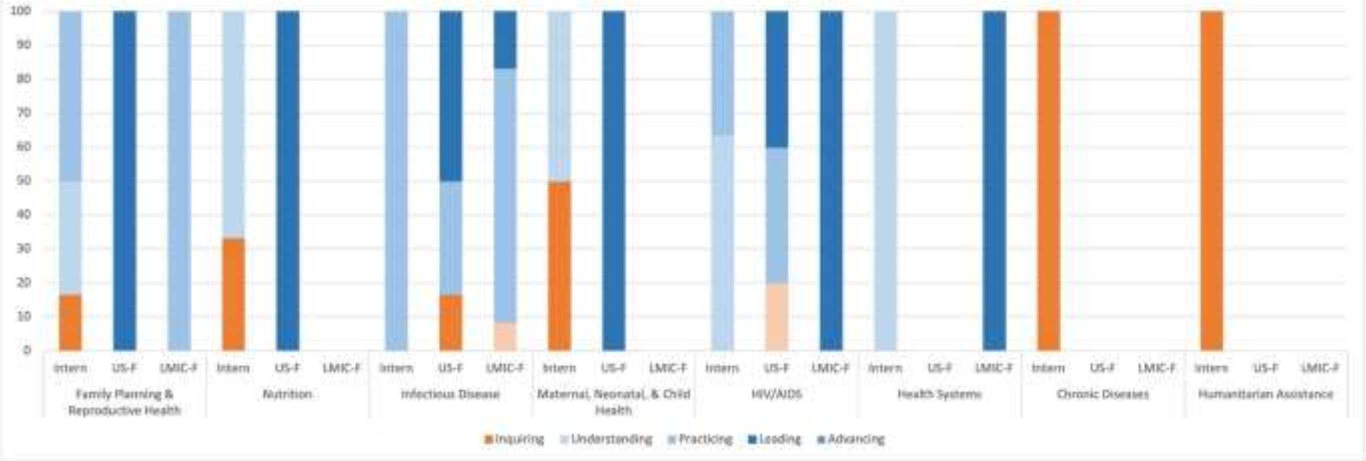

*Shades of orange indicate proportions of STAR participants that had deficits (as defined by the program—minimum of "understanding" level for interns and minimum of "practicing" for fellow) at baseline. Shades of blue indicate adequate skill levels as defined for each type of participant; darker blue indicates higher skill levels, with the darkest blue indicating "advancing" level.
